# Association of systemic inflammatory markers with postoperative arrhythmias in esophageal cancer: a propensity score matching

**DOI:** 10.1186/s13019-024-02630-0

**Published:** 2024-03-19

**Authors:** Hongbi Xiao, Xiaoxia lv, Siding Zhou, Qinglin Ren, Ziang Zhang, Xiaolin Wang

**Affiliations:** 1 Yangzhou University of Medicine, Yangzhou, China; 2 Department of Thoracic Surgery, Northern Jiangsu People’s Hospital, Yangzhou, China

**Keywords:** Postoperative arrhythmia, Systemic immune inflammation index (SII), Esophagectomy, Neutrophil-to-lymphocyte ratio (NLR), Propensity score matching (PSM)

## Abstract

**Background:**

The severity and prognosis of an array of inflammatory diseases have been predicted using systemic inflammatory indices, such as the neutrophil-to-lymphocyte ratio (NLR), platelet-to-lymphocyte ratio, lymphocyte-to-monocyte ratio (LMR), derived neutrophil-to-lymphocyte ratio (dNLR), and systemic immune inflammation index (SII). The purpose of this study was to examine the association between systemic inflammatory markers and postoperative arrhythmias (PA) in esophageal cancer patients.

**Methods:**

In the study, laboratory-related parameters were gathered and examined in 278 patients (non-PA = 221, PA = 57). Fit separate propensity score matching (PSM) within subgroup strata (surgery approaches); match within strata, and aggregate for main analysis. Finally, we established a 1:1(57:57) model. The ability of inflammatory makers on the first post-esophagectomy day to distinguish PA from postoperative non-arrhythmia (non-PA) by receiver operating characteristic (ROC) analysis.

**Results:**

On the first post-esophagectomy day, there was a greater difference between PA and non-PA in terms of white blood cell (WBC) and neutrophil (NE), Neutrophil percentage (NE%), NLR, dNLR, LMR, and SII. After PSM, the following variables were substantially different between non-PA and PA: NE%, NLR, dNLR, and SII. It was found that WBC, NE, NE%, NLR, dNLR, LMR, and SII had the area under the curve (AUC) that was higher than 0.500 in ROC analysis, with NLR and SII having the highest AUC (AUC = 0.661). The indicators were subjected to binary logistic regression analysis, which increased the indicators' predictive ability (AUC = 0.707, sensitivity = 0.877).

**Conclusion:**

On the first post-esophagectomy day, systemic inflammatory indicators were significantly correlated with both PA and non-PA, and high SII and NLR are reliable markers of PA.

**Supplementary Information:**

The online version contains supplementary material available at 10.1186/s13019-024-02630-0.

## Introduction

One of the most common cancers and the sixth largest cause of cancer-related death worldwide is esophageal cancer [1]. Surgery is still the preferred course of treatment for people with removable esophageal cancer, however, one of the most frequent side effects of general thoracic surgery is PA. According to the majority of studies, the likelihood of developing new arrhythmia after esophagectomy ranges from 9 to 46% [2–4]. Although PA are typically short-lived, they can occasionally lead to more significant issues like cardiac failure, hemodynamic instability, and thromboembolic complications. In addition, PA has been linked to higher postoperative mortality, longer hospital stays, and higher hospital expenses [5]. We reviewed the arrhythmia literature and found that the following factors are linked to PA [2, 6, 7]: age, gender, history of smoking, history of hypertension, history of peripheral vascular disease, history of cardiac stenting or angina pectoris, preoperative pulmonary infection, preoperative left heart diastolic insufficiency, surgical approach, prolonged mechanical ventilation, and location of the lesion.

Atrial fibrillation following cardiac surgery has been linked to inflammation and oxidative stress, according to a study [8]. By estimating a patient's risk of different outcomes, inflammation-related metrics, and scores have recently become very popular in helping doctors make judgments. A few biomarkers that can predict systemic inflammation include the NLR, PLR, LMR, SII, and dNLR. Because they are readily available markers that can be determined from a straightforward blood count and exhibit predictive importance for disease and outcome, these indicators of inflammation have recently gained attention.

In esophageal surgery, the relationship between perioperative-related inflammatory markers and PA has not been investigated. In the present study, 278 postoperative esophageal patients were examined, 57 of whom developed PA. We collected demographically relevant characteristics of patients, and surgery-related factors, and performed PSM to explore the relationship between perioperative-related inflammatory markers and the occurrence of PA.

## Methods

**Fig. 1:**
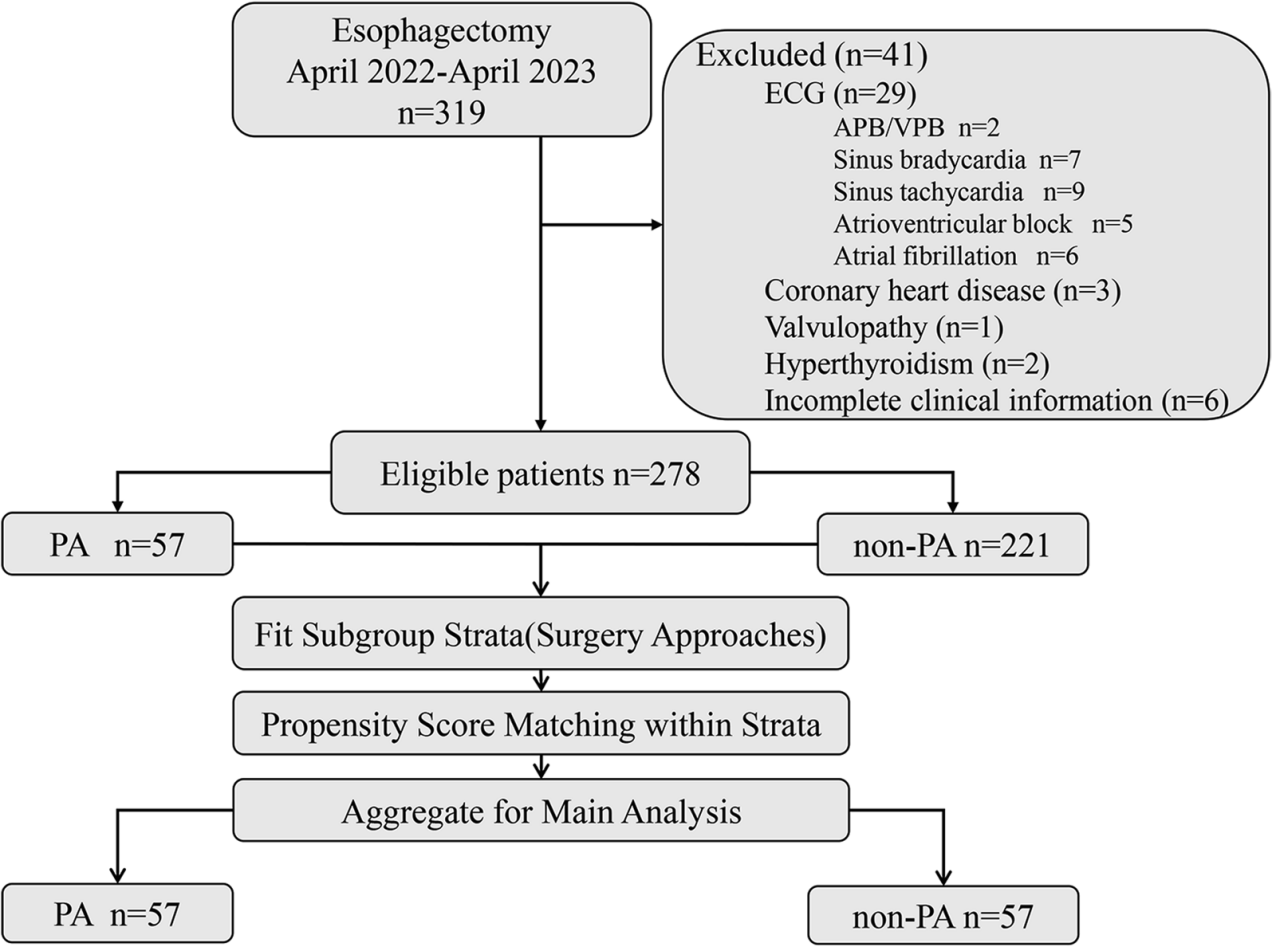
Flowchart of patients' inclusion and exclusion criteria. ECG: electrocardiograph. APB: atrial premature beat. VPB: ventricular premature beat

We examined clinical information from our hospital's electronic medical record system on patients who underwent removable radical esophageal cancer surgery between April 2021 and April 2023(Fig. 1). Minimally invasive surgery is performed with McKeown minimally invasive esophagogastric resection via the right thoracic approach. Open surgery is performed with open Ivor Lewis esophagogastric resection via the right thoracic approach.

Inclusion and exclusion criteria

To establish if it was appropriate to include patients, we created the following eligibility standards:

1. An operable esophageal cancer was chosen as the target disease.
2. PA such as atrial fibrillation, atrial flutter, supraventricular tachycardia, ventricular tachycardia, and ventricular fibrillation, are identified by cardiac monitoring or routine electrocardiograms.
3. Disqualifying anyone with concurrent or prior concurrent other malignancies
4. Discarding patients with malignancies at the esophageal-gastric junction
5. Discarding people who have had thyroid issues in the past
6. Discarding patients with abnormal preoperative electrocardiograms or those who have had heart conditions in the past.
7. Patients who were lost to follow-up were not taken into account in order to guarantee the accuracy and objectivity of the data gathered.

### Propensity score matching (PSM)

To balance potential differences in baseline characteristics and reduce selection bias, PSM was selected for analysis. To avoid differences due to different surgery approaches, we fit separate PSM within unique combinations of subgroup strata (surgery approaches) and use a 1:1 optimal matching algorithm to match PA and non-PA in surgery approaches, and aggregate for the main analysis [9]. PA was used as the independent variable, and the following covariates: gender, left ventricular ejection fraction (LVEF), BMI, pulmonary function FEV1/FVC, American Society of Anesthesiologists Physical Status Classification System (ASA classification), smoking history, history of hypertension, history of diabetes, location of esophageal tumor, clinical TNM stage, length of operation, blood loss, and history of neoadjuvant therapy were used as covariates. The variables were chosen based on the well-known and significant risk factors for arrhythmia following esophagectomy for malignancy. The statistical software used was R 4.3.0 open-source software ( http://www.R-project.org; “MatchIt” and “optmatch” packages).

### Statistical analysis

R 4.3.0 open-source software and IBM SPSS Statistics 26.0 were both used to conduct the statistical analysis. Continuous normally distributed data were evaluated using a student's unpaired two-tailed t-test in the original data analysis. To evaluate continuous, non-normally distributed data, the Wilcoxon rank-sum test was applied. Data with noncontinuous (categorical variables) were examined using Fisher's exact tests or chi-square testing. Following PSM, student-paired two-tailed t-tests were used to assess continuously distributed normally distributed data. The Wilcoxon paired rank sum test was used to evaluate continuous, non-normally distributed data. Discontinuous (categorical variable) data were analyzed using the paired chi-square test or Fisher's exact test. Statistics were considered significant for *p*-values < 0.05. Normally distributed variables were denoted by mean and standard deviation (SD), whereas abnormally distributed variables were denoted by median and IQR (interquartile range) values. The variables with significant differences in inflammatory markers on the first post-esophagectomy day were examined using a binary logistic regression model. To assess the accuracy of the risk of PA prediction, the ROC and AUC were computed. The correct cutoff values for the inflammation index were obtained using the Youden index.

We were unable to secure participants' written agreement for our study because it was retrospectively planned. Our study's protocol met with the Declaration of Helsinki's guiding principles and was approved by the regional ethics council.

## Result

### Patient characteristics

Between April 2021 and April 2023, 319 patients received transthoracic esophagectomy at our facility. A total of 278 patients, including 57 patients with PA and 221 patients without PA, were enrolled in the study in accordance with the inclusion and exclusion criteria. In our study, preoperative hemorrhage and age differed statistically significantly. In order to explore whether there are differences in inflammatory markers between arrhythmia and non-arrhythmia after esophageal cancer surgery, we performed correlational analyses. The baseline disease-related characteristics of the patients are shown in Table 1.

**Table 1:** Patient and treatment-related characteristics in relation to surgical procedure

Characteristic	Before matching		*p*-value	After matching		*p*-value
	PA n = 57	Non-PA		PA	Non-PA	
		n = 221		n = 57	n = 57	
Gender (n)			0.299			0.548
Male	37	159		37	40	
Female	20	62		20	17	
Age (yr)			0.002			0.700
< 65	23	46		23	21	
≥ 65	34	175		34	36	
LVEF (%)	61.00 [60.00,62.00]	61.00 [60.00,63.00]	0.570	60.95 [59.50,62.50]	60.74[59.24,62.95]	0.889
BMI (kg/m^2^)	23.94 (± 3.28)	23.61 (± 3.05)	0.473	23.94 (± 3.28)	23.84 (± 2.85)	0.850
FEV1/FVC	82.16 [76.57,87.35]	78.80 [74.09,84.46]	0.054	82.16 [76.58,87.32]	81.63[76.32,87.11]	0.923
ASA classification (n)			0.878			1.000
I	22	81		22	21	
II	35	140		35	36	
Smoking (n)	19	87	0.403	19	22	0.558
Level of tumor (n)			0.097			0.971
Upper 1/3	1	16		1	1	
Middle 1/3	46	152		46	45	
Lower 1/3	10	53		10	11	
Hypertension (n)	17	78	0.438	17	18	1.000
Diabetes (n)	5	15	0.818	5	6	0.751
Neoadjuvant (n)	5	20	0.948	5	4	0.728
Surgery approaches (n)			0.060			1.000
MIE/Mckenown	13	80		13	13	
OE/Ivor Lewis	44	141		44	44	
Blood loss (ml)	200.00 [100.00,225.00]	200.00 [100.00,200.00]	0.012	194.44 [135.94,247.22]	179.17[115.48,238.54]	0.447
Length of operation (min)	225.00 [205.00,250.00]	215.00 [190.00,247.50]	0.116	226.00 [205.25,248.50]	228.33[197.50,266.25]	0.557
c TNM (n)			0.351			0.888
0	5	18		5	6	
I	11	57		11	10	
II	26	110		26	29	
III	15	36		15	15	

Data are n, median (range), and mean (±SD)

### Clinical features Of 57 patients with PA

A total of 278 individuals were included in our study, and 57 of them developed arrhythmia with a 20.50% incidence rate. It was comparable to prior studies' incidence rates. 30 of the 57 patients with PA had atrial fibrillation, and amiodarone or amiodarone in conjunction with other medications was administered to all patients with atrial fibrillation following the onset of the condition. Amiodarone was administered to three patients in combination with lidocaine, metoprolol, and cediran, respectively. On the second postoperative day, one patient experienced an acute myocardial infarction, which required transfer to the intensive care unit (ICU). On the second postoperative day, one patient experienced a significant cerebral infarction and passed away on the seventh postoperative day. 27 patients experienced sinus tachycardia; 8 of them received metoprolol treatment, while 19 others received no immediate treatment. In our study, 51 (89.47%) PA patients had arrhythmias that developed within the first three days after surgery.

### Laboratory markers

Our skilled nurses collect blood prior to surgery, the first post-esophagectomy day, and the fifth post-esophagectomy day. Absolute white blood cell count (AWC), absolute neutrophil count (ANC), absolute lymphocyte count (ALC), absolute platelet count (APC), absolute monocyte count (AMC), and albumin levels were measured using complete blood counts and biochemical testing. On the first post-esophagectomy day, blood was drawn to check for 12 cytokine collections, including IFN-α, IFN-γ, TNF-α, IL-1β, IL-2, IL-4, IL-5, IL-6, IL-8, IL-10, IL-12, and IL-17.

### Study on the correlation between inflammatory markers and arrhythmia

Inflammation-related scores were calculated as follows: NLR = ANC/ALC, PLR = APC/ALC, LMR = ALC/AMC, SII = ANC × APC/ALC, dNLR = ANC/(AWC-ANC), and NE% = ANC/AWC × 100, PNI = 10 × albumin(g/dl) + 0.005 × ALC.

**Fig. 2:**
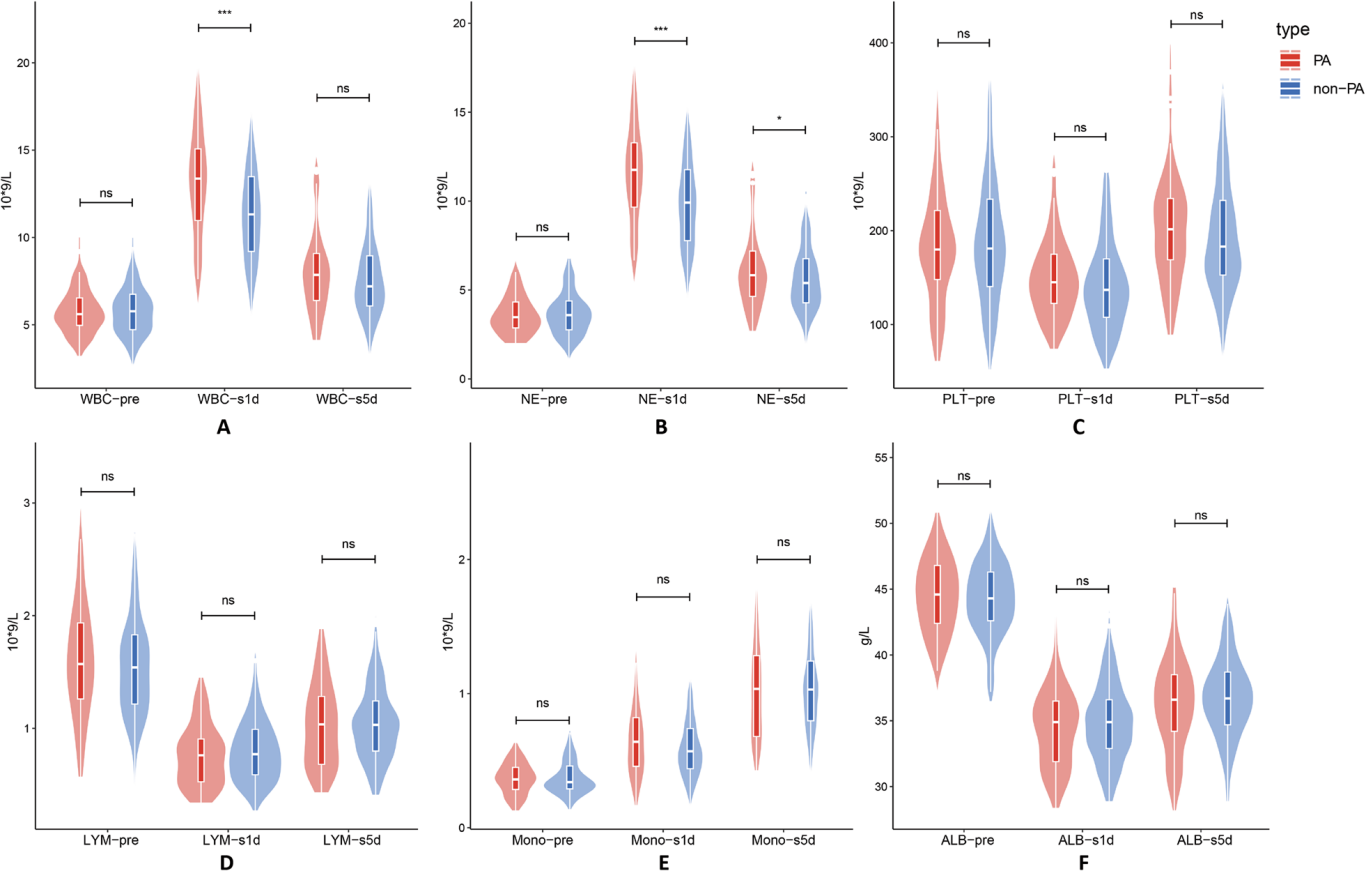
The inflammatory laboratory results and distribution variations between the PA and non-PA groups were examined before surgery, On the first and fifth post-esophagectomy day. **A** WBC (white blood cell); **B** NE (neutrophil); **C** PLT (platelet); **D** LYM (lymphocyte); **E** Mono (monocyte); **F** ALB (albumin). *P*-value: ns > 0.05, * ≤ 0.05, ** ≤ 0.01, *** ≤ 0.001

**Fig. 3:**
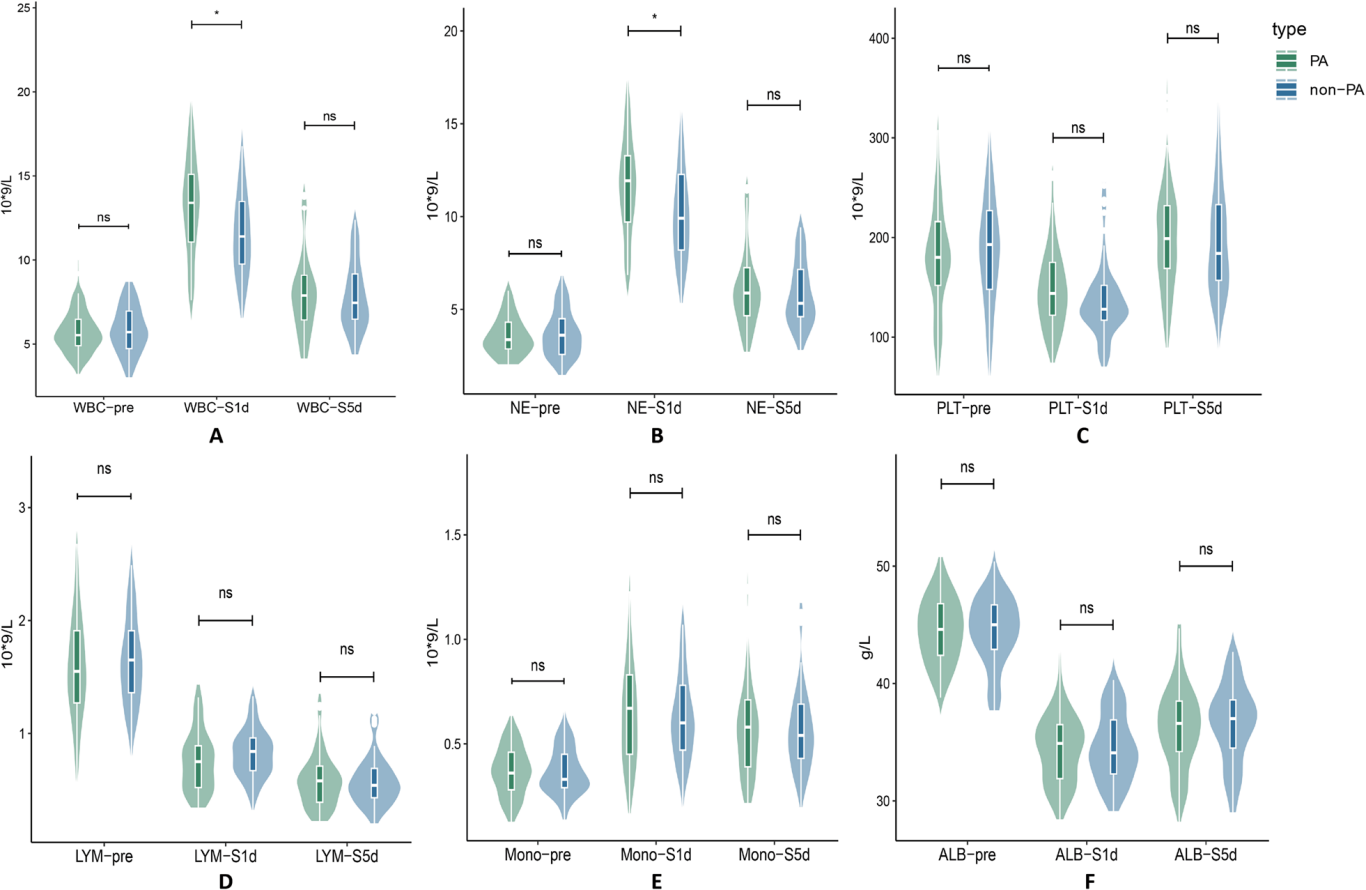
Following PSM, the inflammatory laboratory results and distribution variations between the PA and non-PA groups were examined before surgery, On the first and fifth post-esophagectomy day. *p*-value: ns > 0.05, * ≤ 0.05, ** ≤ 0.01, *** ≤ 0.001

The pertinent laboratory analysis data of the research subjects are shown in Table 2, Fig. 2. There was no significant difference in baseline inflammatory values between PA and non-PA patients. Lymphocyte counts, platelet counts, and albumin levels all fell to varied degrees on the first post-esophagectomy day among the biomarkers, but there were no appreciable variations between PA and non-PA. Both the NE (11.60 vs. 10.04, *p*-value = 0.001) and the WBC (13.11 vs. 11.52, *p*-value = 0.001) significantly varied between the two groups. Additionally, there were variations in the growth of NE (7.97 vs. 6.26, *p*-value < 0.001), monocytes (0.37 vs. 0.21, *p*-value = 0.017), and WBC (7.33 vs. 5.62, *p*-value < 0.001). All biomarkers gradually returned to normal levels on the fifth post-esophagectomy day, but differences in NE (6.50 vs. 5.69, *p*-value = 0.046) persisted. There was no significant difference in other indicators between the two groups after PSM (Fig. 3), with the exception of WBC (13.11 vs. 11.91, *p*-value = 0.041) and NE (11.60 vs. 10.35, *p*-value = 0.024) on the first post-esophagectomy day.

**Table 2:** Perioperative inflammatory indicators

Indicators	Before matching		*p*-value	After matching		*p*-value
	PA n = 57	Non-PA n = 211		PA n = 57	Non-PA n = 57	
WBC pre	5.78 (± 1.36)	5.90 (± 1.57)	0.601	5.78 (± 1.36)	5.85 (± 1.43)	0.759
WBC 1d	13.11 (± 3.28)	11.52 (± 3.22)	0.001	13.11 (± 3.28)	11.91 (± 3.19)	0.043
WBC 5d	8.38 (± 3.06)	7.64 (± 2.27)	0.093	8.38 (± 3.06)	7.95 (± 2.40)	0.386
d-value(WBC1d)	7.33 (± 3.03)	5.62 (± 2.89)	< 0.001	7.33 (± 3.03)	6.05 (± 3.05)	0.029
NE pre	3.63 (± 1.14)	3.77 (± 1.42)	0.470	3.63 (± 1.14)	3.70 (± 1.29)	0.726
NE 1d	11.60 (± 3.00)	10.04 (± 3.04)	0.001	11.60 (± 3.00)	10.35 (± 3.01)	0.024
NE 5d	6.50 (± 2.85)	5.69 (± 1.94)	0.046	6.50 (± 2.85)	5.95 (± 2.03)	0.221
d-value(NE 1d)	7.97 (± 2.80)	6.26 (± 2.79)	< 0.001	7.97 (± 2.80)	6.65 (± 2.86)	0.015
PLT pre	185.75 (± 68.53)	188.04 (± 68.21)	0.822	185.75 (± 68.53)	187.61 (± 60.56)	0.868
PLT1d	152.77 (± 47.91)	142.59 (± 48.30)	0.156	152.77 (± 47.91)	139.32 (± 41.98)	0.137
PLT5d	203.25 (± 64.64)	194.67 (± 61.60)	0.355	203.25 (± 64.64)	198.02 (± 59.66)	0.676
d-value(PLT 1d)	− 32.98 (± 42.87)	− 45.45 (± 42.90)	0.051	− 32.98 (± 42.87)	− 48.30 (± 40.98)	0.024
LYM pre	1.61 (± 0.53)	1.56 (± 0.51)	0.575	1.61 (± 0.53)	1.64 (± 0.43)	0.671
LYM 1d	0.76 (± 0.27)	0.81 (± 0.30)	0.232	0.76 (± 0.27)	0.84 (± 0.28)	0.087
LYM 5d	1.02 (± 0.37)	1.06 (± 0.37)	0.449	1.02 (± 0.37)	1.07 (± 0.37)	0.527
d-value(LYM 1d)	− 0.85 (± 0.41)	− 0.75 (± 0.43)	0.134	− 0.85 (± 0.41)	− 0.79 (± 0.40)	0.484
Mono pre	0.36 (± 0.12)	0.38 (± 0.17)	0.469	0.36 (± 0.12)	0.36 (± 0.11)	0.983
Mono 1d	0.68 (± 0.33)	0.60 (± 0.24)	0.075	0.68 (± 0.33)	0.63 (± 0.22)	0.275
Mono 5d	0.59 (± 0.25)	0.57 (± 0.21)	0.560	0.59 (± 0.25)	0.58 (± 0.22)	0.847
d-value(Mono 1d)	0.37 (± 0.29)	0.21 (± 0.23)	0.017	0.37 (± 0.29)	0.26 (± 0.19)	0.230
ALB pre	44.32 (± 3.30)	44.29 (± 2.98)	0.960	44.32 (± 3.30)	44.60 (± 3.30)	0.661
ALB 1d	34.48 (± 3.08)	34.78 (± 3.76)	0.583	34.48 (± 3.08)	34.65 (± 3.13)	0.755
ALB 5d	36.25 (± 3.74)	36.73 (± 3.18)	0.322	36.25 (± 3.74)	36.67 (± 3.68)	0.543
d-value(ALB 1d)	− 9.83 (± 3.23)	− 9.51 (± 4.43)	0.610	− 9.83 (± 3.23)	− 10.48 (± 5.98)	0.431

Mean (± SD); d-value (WBC, NE, PLT, LYM, Mono, ALB): the first post-esophagectomy day and preoperative differential value of white blood cell, neutrophil (NE), platelet (PLT), lymphocyte (LYM), monocyte(mono), albumin (ALB)

**Fig. 4:**
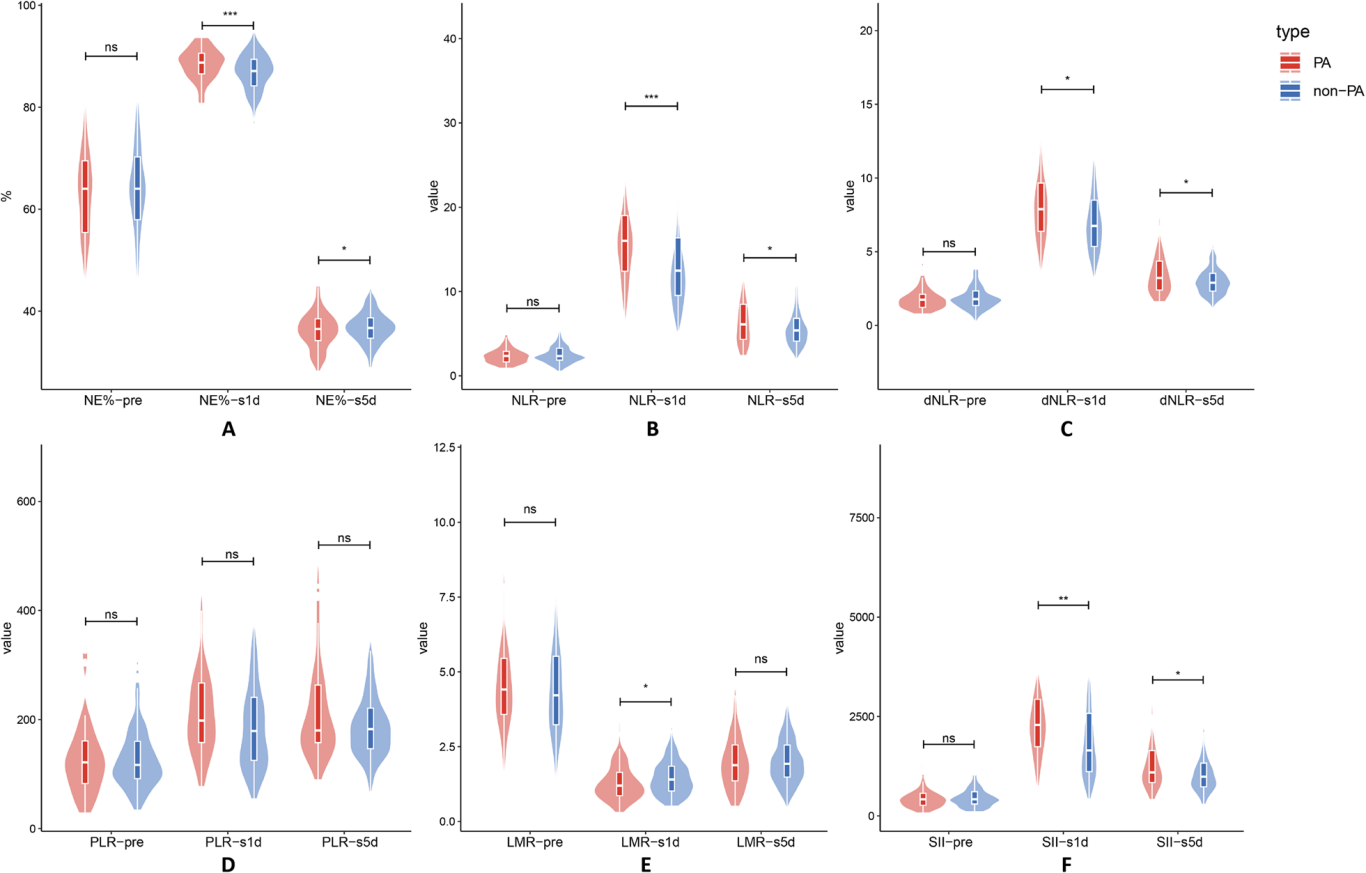
The inflammatory scores and distribution between the PA group and the non-PA group were examined before surgery, On the first and fifth post-esophagectomy day. *p*-value: ns > 0.05, * ≤ 0.05, ** ≤ 0.01, *** ≤ 0.001

**Fig. 5:**
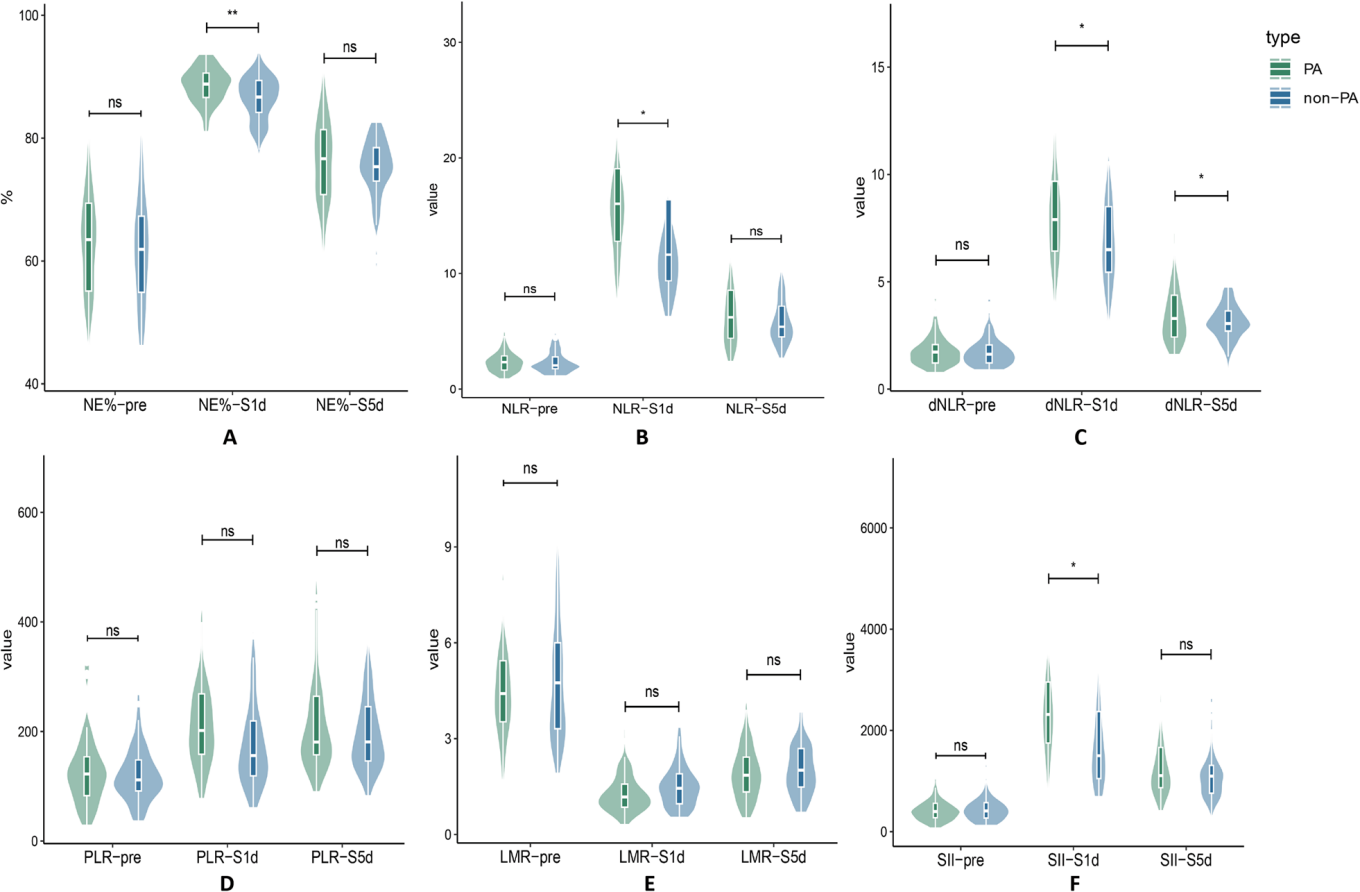
Following PSM, the inflammatory scores and distribution between the PA group and the non-PA group were examined before surgery, On the first and fifth post-esophagectomy day. *p*-value: ns > 0.05, * ≤ 0.05, ** ≤ 0.01, *** ≤ 0.001

The estimated values of the pertinent inflammatory indicators are shown in Table 3, Fig. 4. Preoperative inflammatory indexes did not significantly differ between the two groups. On the first post-esophagectomy day, inflammatory markers, such as NE% (88.52 vs. 86.56, *P*-value < 0.001), NLR (16.75 vs. 13.77, *P*-value = 0.001), dNLR (8.29 vs. 7.22, *p* = 0.011), LMR (1.29 vs. 1.49, *p*-value = 0.034), and SII (2533.16 vs. 1977.74, *p*-value = 0.002) were found to be significantly associated with PA. On the fifth post-esophagectomy day, NE% (76.23 vs. 73.67, *p* = 0.011), NLR (7.06 vs. 5.79, *p* = 0.018), dNLR (3.64 vs. 3.04, *p* = 0.013), and SII (1398.19 vs 1111.33, *p*-value = 0.031) all showed higher inflammatory markers. Preoperative inflammatory markers showed no overt abnormalities after PSM (Fig. 5). On the first post-esophagectomy day, there was a significant difference in the following variables: NE% (88.52 vs. 86.24, *p*-value = 0.001), NLR (16.75 vs. 13.40, *p*-value = 0.004), dNLR (8.29 vs. 7.18, *p* = 0.024) and SII (2533.16 vs. 1901.52, *p*-value = 0.013). On the fifth post-esophagectomy day, dNLR (3.64 vs.3.09, *p*-value = 0.033) was significantly different between the two groups.

**Table 3:** Perioperative nutritional indicators

Indicators	Before matching		*p*-value	After matching		*p*-value
	PA n = 57	Non-PA n = 211		PA n = 57	Non-PA n = 57	
NE% pre	62.82 (± 8.71)	63.19 (± 9.91)	0.797	62.82 (± 8.71)	61.97 (± 8.97)	0.556
NE% 1d	88.52 (± 3.01)	86.56 (± 4.19)	< 0.001	88.52 (± 3.01)	86.24 (± 4.26)	0.001
NE% 5d	76.23 (± 6.89)	73.67 (± 6.70)	0.011	76.23 (± 6.89)	74.38 (± 6.36)	0.158
NLR pre	2.52 (± 1.30)	2.71 (± 1.62)	0.422	2.52 (± 1.30)	2.39 (± 1.06)	0.534
NLR 1d	16.75 (± 6.09)	13.77 (± 6.26)	0.001	16.75 (± 6.09)	13.40 (± 5.83)	0.004
NLR 5d	7.06 (± 3.75)	5.79 (± 2.46)	0.018	7.06 (± 3.75)	5.95 (± 2.13)	0.067
dNLR pre	1.81 (± 0.78)	1.94 (± 0.96)	0.339	1.81 (± 0.78)	1.83 (± 0.88)	0.916
dNLR 1d	8.29 (± 2.65)	7.22 (± 2.87)	0.011	8.29 (± 2.65)	7.18 (± 2.72)	0.024
dNLR 5d	3.64 (± 1.67)	3.04 (± 1.08)	0.013	3.64 (± 1.67)	3.09 (± 0.83)	0.033
PLR pre	130.45 (± 68.35)	130.45 (± 59.43)	0.963	130.45 (± 68.35)	121.67 (± 50.84)	0.462
PLR 1d	223.60 (± 101.43)	198.10 (± 99.84)	0.088	223.60 (± 101.43)	184.95 (± 93.34)	0.059
PLR 5d	217.53 (± 91.28)	795.64 (± 72.28)	0.097	217.53 (± 91.28)	199.81 (± 73.65)	0.291
LMR pre	4.69 (± 1.81)	4.53 (± 1.87)	0.552	4.69 (± 1.81)	4.88 (± 1.91)	0.614
LMR 1d	1.29 (± 0.61)	1.49 (± 0.644)	0.034	1.29 (± 0.61)	1.50 (± 0.66)	0.088
LMR 5d	1.97 (± 0.90)	2.02 (± 0.77)	0.653	1.97 (± 0.90)	2.01 (± 0.78)	0.779
SII pre	475.98 (± 313.43)	500.73 (± 317.64)	0.599	475.98 (± 313.43)	454.98 (± 266.15)	0.703
SII 1d	2533.16 (± 1203.04)	1977.74 (± 1174.75)	0.002	2533.16 (± 1203.04)	1901.52 (± 1121.33)	0.013
SII 5d	1398.19 (± 942.14)	1111.33 (± 565.65)	0.031	1398.19 (± 942.14)	1173.88 (± 555.85)	0.147
PNI pre	52.37 (± 4.54)	52.13 (± 4.09)	0.699	52.37 (± 4.54)	52.84 (± 3.88)	0.549
PNI 1d	38.28 (± 3.51)	38.85 (± 4.24)	0.357	38.28 (± 3.51)	38.90 (± 3.75)	0.312
PNI 5d	41.37 (± 4.62)	42.07 (± 4.20)	0.275	41.37 (± 4.62)	42.02 (± 4.59)	0.477

Data are n, median (range), and mean (± SD)

In recent years, minimally invasive esophagectomy with the goal of reducing surgical stress has been widely performed. In this study, open esophagectomy accounted for the majority, in order to exclude whether the different Surgery approaches affected the postoperative inflammatory indicators. The inflammatory indicators on the first day after surgery were analyzed in the raw data between the surgical method and the surgical method (Additional file 1:  Table S1). In the raw data, except for monocytes, which differed according to the surgical method, the other indicators showed no difference.

**Fig. 6:**
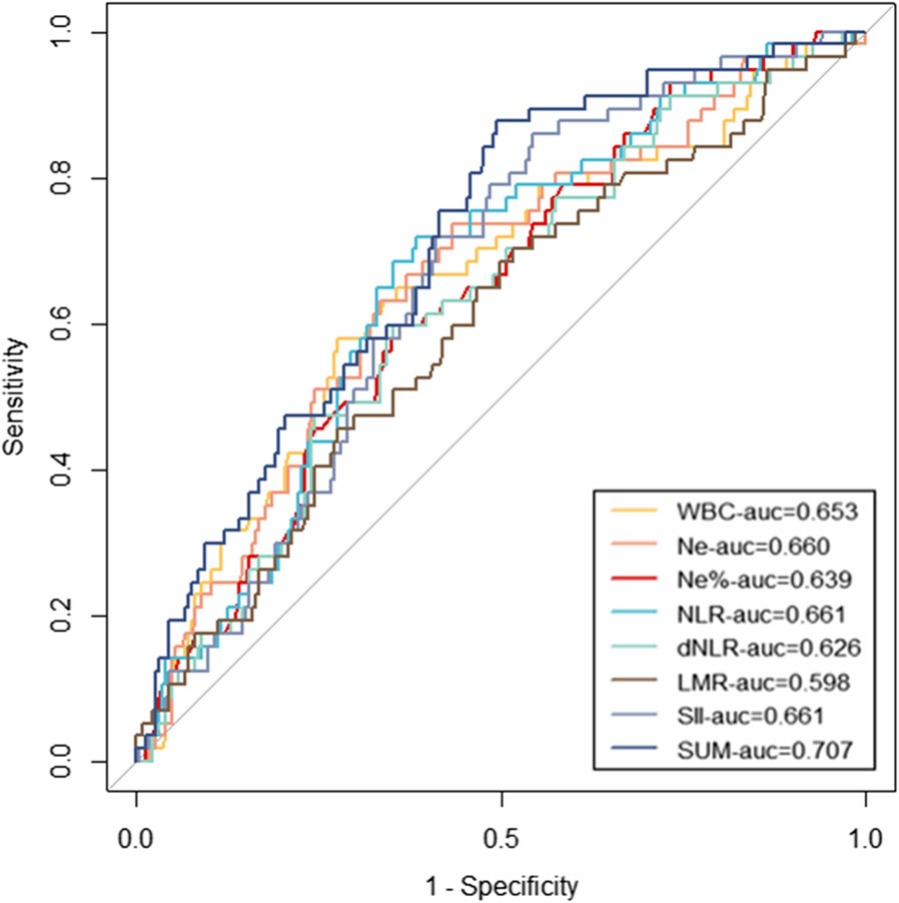
Receiver operating characteristics curve for WBC, Ne, Ne%, NLR, dNLR, LMR, and SII

The estimated PA postoperative first-day inflammatory index's predictive power is indicated by the ROC and AUC curves in Table 4 and Fig. 6. On the first post-esophagectomy day, the AUC of WBC, NE, NE%, NLR, dNLR, LMR, and SII was higher than 0.500 on the original data; NLR and SII had the highest area (AUC = 0.661) among the test results. LMR showed the best specificity (95.5%), however, it also had a low sensitivity. The sensitivity of SII, which was the highest, had a value of 86.0%. SUM derived from binary logistic regression analysis improved prediction (AUC = 0.707, specificity = 0.507, sensitivity = 0.877). However, there was a lack of statistical significance between the SUM and the biggest AUC values (SII and NLR) in the predictive power (*p*-value = 0.119).

**Table 4:** ROC plot of the optimal inflammatory scores

Inflammatory scores	AUC	95%CI	Cut-off	Specificity	Sensitivity
NLR 1d	0.661	0.585–0.736	13.684	0.615	0.719
dNLR 1d	0.626	0.548–0.705	7.582	0.647	0.696
NE% 1d	0.639	0.562–0.716	88.350	0.647	0.596
LMR 1d	0.598	0.515–0.681	0.921	0.955	0.158
SII 1d	0.661	0.589–0.733	1525.934	0.457	0.860
WBC 1d	0.653	0.572–0.735	13.005	0.724	0.579
NE 1d	0.660	0.581–0.739	10.200	0.566	0.737
SUM	0.707	0.636–0.778	–	0.507	0.877

ROC—receiver operating characteristic; AUC—area under curve; CI—confidence interval

### Study on the correlation between cytokines and arrhythmia

An essential part of the systemic inflammatory response is played by cytokines. It is widely known that surgical trauma causes the release of mediators involved in the acute-phase response, including interleukin-1 and IL-6 [10]. In our study, there were no discernible differences in postoperative arrhythmic and non-arrhythmic patients' levels of IFN-α, IFN-γ, TNF-α, IL-1β, IL-2, IL-4, IL-5, IL-6, IL-8, IL-10, IL-12, and IL-17. After PSM, a lack of statistically significant variations between the two groups was possibly seen (Additional file 2: Table S2).

### Correlation between postoperative outcomes and arrhythmia

Patients with PA had longer hospital stays than patients without PA (IQR:13 vs. 12; *p*-value 0.001). Between the two groups, there was no discernible difference in the frequency of postoperative analgesic usage. Other perioperative outcomes were more common in patients with arrhythmia than in those without (*p* = 0.002) (Table 5). Complications were not statistically compared individually in our study because the sample size was limited and the incidence of postoperative complications was low. The specific problems were as follows: PA developed in 3 cases of postoperative pulmonary embolism, 8 cases of respiratory failure, one case of acute cerebral infarction, and 2 cases of hemothorax. 4 (66.6%) of the 6 patients with anastomotic leakage went on to develop PA. 5 (20.8%) of the 24 pleural effusion patients went on to develop PA. 4 (36.3%) of the 11 pneumonia patients went on to develop PA. 1 (33.3%) of the 3 chylothorax patients went on to develop PA. 2 (50.0%) of the 4 pneumothorax patients went on to develop PA. Patients who had pyloric obstruction (3 cases) and surgical incision infection (4 cases) did not get PA.

**Table 5:** Perioperative outcomes

outcomes		PA n = 57	Non-PA n = 221	*p*-value
Frequency of analgesics		2.00[1.00,3.00]	2.00[1.00,3.00]	0.553
Length of stay(day)		13.00[11.50,15.50]	12.00[11.00,13.00]	< 0.001
Complication(n)				
Yes		20	37	0.002
No		37	184	

Data are n, median [inter-quartile range, IQR], and mean (± SD)

## Discussion

High surgical stress and a high incidence of postoperative arrhythmia are associated with esophagectomy. All organs may be affected and the secretion of numerous proinflammatory and antiinflammatory substances may increase as a result of the numerous local or systemic inflammatory reactions it can set off. The immune system's delicate equilibrium is disturbed by cancer surgery. Significant surgical trauma may lower the survival rate of cancer patients and increase postoperative adverse responses [11].

Recent research has revealed a link between systemic indicators of inflammation and a poor prognosis for esophageal cancer. For instance, in esophageal cancer patients, the level of C-reactive protein indicates prognosis [12]. However, not all hospitals frequently conduct C-reactive protein testing. The information regarding red blood cells, white blood cells, and platelets is available through a complete blood count, which is simple to administer, less expensive, and easily accessible. Platelets release pro-inflammatory mediators such as desmoplasticization and cytokines. Platelet activation plays an important role in coronary artery disease (CAD) and cardiovascular disease (CVD) [13, 14]. A strong predictor of death in patients with acute myocardial infarction is a high baseline platelet count [15]. A common indicator of inflammation in CVD is the WBC count. Abdelhadi RH et al. showed that there was a strong correlation between elevated white blood cells after coronary artery bypass grafting or heart valve surgery and the occurrence of atrial fibrillation after cardiac surgery, supporting a role in the mechanism of atrial fibrillation after cardiac surgery [16]. Lymphocytes are involved in the long-term response of the immune system, and the cell-mediated immune response is largely dependent on lymphocytes; large numbers of infiltrating lymphocytes are associated with a favorable prognosis, whereas lymphopenia is considered to be a predictor of a poor prognosis [17]. After major surgery, lymphocyte numbers and function are known to continuously drop [18]. By preventing lymphocyte-mediated cytolysis, neutrophils can provide a tumor-friendly environment. One of the most significant innate immune system mediators, the neutrophils are abnormally overactivated by a number of inflammatory cytokines and chemokines produced by cancer cells, which can enhance cell arrest within capillaries and lead to the destruction of healthy host tissues [19]. Increased neutrophil counts indicate a higher risk of negative outcomes in percutaneous coronary intervention (PCI) patients [20]. Similar to this, alterations in inflammatory markers roughly coincide with the time course of atrial fibrillation following heart surgery [21].

Numerous systemic indicators of inflammation have been identified to aid in the diagnosis and development of numerous illnesses, including inflammatory diseases. Integration of various immune pathways, including NLR, dNLR, PLR, LMR, and SII, which are interdependent and play a significant role in prognosis, in COVID-19 [22], preterm labor [23], acute pancreatitis [24], rheumatic diseases [25], and coronary artery disease [14, 26–28]. These indices gather various complete blood count values and are more prone to react to inflammation in afflicted people. Since chronic rather than acute inflammation is largely mediated by monocytes, which are monocyte-derived macrophages, and lymphocytes, LMR has been employed as a marker of the chronic systemic inflammatory response. Increased neutrophil and platelet counts are characteristic changes in acute systemic inflammatory responses. NLR, dNLR, and PLR have been reported to be associated with the development of cardiovascular disease, and several studies have demonstrated that high NLR and high mean platelet volume (MPV) are independent predictors of long-term major adverse cardiovascular events (MACE) after PCI, especially in acute coronary syndrome (ACS) [26]. Other studies have also shown that PLR at admission is significantly associated with the severity and complexity of coronary atherosclerosis in patients with ACS [13, 28]. In this study, systemic inflammatory indices such WBC, NE, NE%, NLR, dNLR, LMR, and SII on the first post-esophagectomy day were considerably greater in patients with PA than in patients with non-PA, and LMR was significantly lower than that in non-PA. Among of all the inflammatory indices, SII and NLR exhibited the highest area under the curve (AUC = 0.661). LMR showed the best specificity (95.5%), however it also had a low sensitivity. With a sensitivity of 86.0%, SII had the highest level, but it was not extremely sensitive.

In recent years, the SII, SII = ANC × APC/ALC, which incorporates three different types of inflammatory cells and is based on platelet count and NLR, has been established. It considers the inflammatory and immune condition of the patient. SII is a comprehensive measure of inflammation that is highly predictive of cardiovascular disease. High SII readings have been shown to negatively and independently affect the advancement of coronary atherosclerotic plaque, unfavorable progression such as congestive heart failure, hospitalization, and the long-term course of severe coronary syndromes [27, 29, 30]. In another study conducted by Erdoğan et al., it has been found that SII can be a strong predictor of coronary artery occlusion compared to PLR and NLR, which is considered to be hemodynamically significant and can be used as an independent predictor of coronary artery occlusion, which may lead to heart attack [31]. A different study found that SII had a higher level of predictive accuracy than PLR and NLR and could independently predict the presence of left ventricular hypertrophy (LVH) [32]. Additionally, SII may support the differential diagnosis of venous thrombosis patients. It is more accurate than PLR and NLR for estimating venous thrombosis in patients [33]. In addition, on the fifth post-esophagectomy day, some systemic inflammatory indices in our study remained greater in PA patients than in non-PA patients. The recovery of PA patients was slower in terms of systemic nutritional markers.

Surgery-related trauma and the inflammatory reactions that follow are crucial in the etiology of PA. A recent study found that postoperative triggers operating on the delicate atrial substrate created by preoperative, medically induced, and postoperative remodeling processes are what cause atrial fibrillation (AF). The addition of transient surgically induced AF-promoting changes to prior atrial remodeling (preexisting substrate) exceeds the threshold vulnerability, allowing autonomic nervous system (ANS)-promoted triggering, inflammation, and oxidative stress to initiate postoperative AF [3]. Research is being done on the pathophysiological pathways that involve oxidative stress and inflammation. Given that arrhythmia may contribute to the development of inflammation and that inflammation may cause arrhythmia. As a result, the two could create a dangerous vicious circle [4]. Along with the intrusiveness of the surgery itself, cytokines that promote inflammation are also known to be released as a result of postoperative infectious complications (PIC) [34]. Recent research has demonstrated that PIC following esophagectomy negatively affects patient survival [35].

According to a study by David Amar and colleagues, taking statins prior to major thoracic surgery was linked to a lower incidence of atrial fibrillation following the procedure [36]. Additionally, James et al. demonstrated that prophylactic intravenous amiodarone was linked to postoperative hypotension, bradycardia, and a longer QTc interval in addition to a lower incidence of AF after esophagectomy [37]. A study showed, early initiation of vasopressin therapy in critically ill adult patients with infectious shock reduced the incidence of new arrhythmias. And there was a trend toward improved cardiac biomarkers in the early pressor group [38]. A substantial association between PA patients and other postoperative problems was found in our study. PA may have been viewed thus far as an early warning indication of other (infectious) issues rather than as the cause of these complications, however, this may be owing to uncertainty in this relatively small study. The prevention of PA may merely serve to conceal early clinical indications of additional surgical problems and to postpone treatment. Because PA is rather simple to cure and typically goes away quickly after the start of medication, the need for prophylaxis of the condition is still debatable. The discovery of risk variables, however, might signal the emergence of more severe issues. Therefore, in order to avoid postoperative arrhythmia surgeons should carefully consider the use of pertinent drugs.

The clinical implications of this study are significant. It is possible to identify early patients who are at high risk of developing PA after esophageal surgery by performing a simple and cost-effective peripheral blood test, which may be easily brought to the attention of surgeons in the perioperative phase. This study has certain limitations. To start, it can be challenging to prove causality and generalization and control for bias and confounding factors because it is based on a single institution. Second, our small sample size, combined with the low incidence of postoperative complications, resulted in too few patients with complications such as anastomotic leakage in our study. Therefore, there is no detailed classification of postoperative complications. Third, in our study, we used the total operative time to flank the thoracic operation time, and unrecorded intrathoracic operation time may lead to some errors in the results. In addition, our minimally invasive surgery for esophageal cancer is Mckenown, and open surgery is Ivor Lewis. We lack open Mckenow and minimally invasive Ivor Lewis to complement the sample and whether the lack of their inflammatory factors is associated with postoperative arrhythmia. Fourth, because this was a clinical study, it was unable to explore the molecular biological pathways by which inflammatory variables affect the arrhythmia that was being examined.

## Conclusion

On the first post-esophagectomy day for patients with esophageal cancer, inflammation ratings NLR, LMR, and SII were substantially different between non-PA and PA, and SII had the highest prognostic value for patients with esophageal cancer who had arrhythmia. Clinicians should pay close attention to patients who have high inflammation scores on the first post-esophagectomy day, be on the lookout for more serious complications, and intensify treatment and management to reduce the impact of arrhythmia on the patient's postoperative recovery in their future clinical work.

### Supplementary Information

**Additional file 1: Table S1**. Perioperative nutritional indicators.

**Additional file 2: Table S2**. Postoperative cytokines.
