# Supplementary material for: Association of systemic inflammatory markers with postoperative arrhythmias in esophageal cancer: a propensity score matching
Source: J Cardiothorac Surg. 2024 Mar 19;19:142. doi: 10.1186/s13019-024-02630-0 (PMC10949772; doi:10.1186/s13019-024-02630-0)
Supplement: Supplementary file 1 — Additional file 1: Table S1. Perioperative nutritional indicators. [file 13019_2024_2630_MOESM1_ESM.docx]

| Supplementary Table 1. Perioperative nutritional indicators | | | |
| --- | --- | --- | --- |
| Indicators | MIE/Mckenown  n=93 | OE/Ivor Lewis  n=185 | p-value |
| WBC 1d | 12.01(±3.25) | 11.77(±3.31) | 0.564 |
| d-value (WBC) | 6.13(±3.00) | 5.89(±3.00) | 0.533 |
| NE 1d | 10.49(±2.98) | 10.30(±3.14) | 0.635 |
| d-value (NE) | 6.81(±2.79) | 6.51(±2.92) | 0.421 |
| NE% 1d | 86.41(±4.31) | 87.25(±3.90) | 0.106 |
| PLT 1d | 143.63(±51.11) | 145.20(±46.98) | 0.799 |
| Mono 1d | 0.66(±0.27) | 0.59(±0.25) | 0.039 |
| LYM 1d | 0.84(±0.35) | 0.78(±0.26) | 0.107 |
| NLR 1d | 13.99(±6.44) | 14.58(±6.29) | 0.466 |
| dNLR 1d | 7.14(±3.03) | 7.58(±2.76) | 0.231 |
| PLR 1d | 192.27(±91.41) | 208.89(±104.59) | 0.194 |
| SII 1d | 1988.38(±1024.20) | 2143.53(±1278.24) | 0.275 |
| LMR 1d | 1.36(±0.52) | 1.49(±0.69) | 0.115 |
| Data are n, median [inter-quartile range, IQR], and mean (±SD) | | | |
