# Supplementary material for: Association of systemic inflammatory markers with postoperative arrhythmias in esophageal cancer: a propensity score matching
Source: J Cardiothorac Surg. 2024 Mar 19;19:142. doi: 10.1186/s13019-024-02630-0 (PMC10949772; doi:10.1186/s13019-024-02630-0)
Supplement: Supplementary file 2 — Additional file 2: Table S2. Postoperative cytokines. [file 13019_2024_2630_MOESM2_ESM.docx]

| Supplementary Table 2. Postoperative cytokines | | | | | | |
| --- | --- | --- | --- | --- | --- | --- |
| Indicators | Before matching | | p-value | After matching | | p-value |
|  | PA  n=57 | Non-PA  n=211 |  | PA  n=57 | Non-PA  n=57 |  |
| IFN-α | 1.78[1.46,1.86] | 1.78[1.45,1.89] | 0.964 | 1.78[1.46,1.86] | 1.78[1.42,1.85] | 0.648 |
| IFN-γ | 2.33[1.48,5.18] | 2.41[2.06,5.11] | 0.213 | 2.33[1.48,5.18] | 2.54[1.91,5.25] | 0.448 |
| TNF-α | 2.01[1.10,3.64] | 2.30[1.80,4.51] | 0.312 | 2.01[1.10,3.64] | 2.01[1.06,4.10] | 0.934 |
| IL-1β | 7.16[2.07,10.49] | 4.55[2.39,11.54] | 0.747 | 7.16[2.07,10.49] | 8.23[2.31,8.50] | 0.462 |
| IL-2 | 1.14[0.92,1.46] | 1.16[0.91,2.11] | 0.695 | 1.14[0.92,1.46] | 1.09[0.89,1.58] | 0.639 |
| IL-4 | 1.31[0.74,1.62] | 1.57[0.88,1.69] | 0.097 | 1.31[0.74,1.62] | 1.22[0.80,1.58] | 0.822 |
| IL-5 | 1.84[1.37,4.00] | 2.45[1.48,4.40] | 0.123 | 1.84[1.37,4.00] | 2.44[1.46,4.13] | 0.584 |
| IL-6 | 36.59[14.76,86.57] | 39.03[20.36,75.53] | 0.669 | 36.59[14.76,86.57] | 45.76[20.89,83.47] | 0.458 |
| IL-8 | 7.59[2.33,25.51] | 7.89[2.32,29.93] | 0.539 | 7.59[2.33,25.51] | 7.65[2.51,27.07] | 0.799 |
| IL-10 | 1.24[0.87,2.34] | 1.50[1.01,2.64] | 0.144 | 1.24[0.87,2.34] | 1.49[1.00,2.66] | 0.158 |
| IL-12 | 1.63[1.14,3.48] | 1.79[1.17,2.53] | 0.482 | 1.63[1.14,3.48] | 1.73[1.03,2.25] | 0.846 |
| IL-17 | 3.65[1.68,11.53] | 4.41[2.01,13.89] | 0.514 | 3.65[1.68,11.53] | 4.72[2.04,13.68] | 0.736 |
| Data are n, median (range), and mean (±SD) | | | | | | |
